# Work addiction, emotional dysregulation, addictive eating, and physical health among full-time employees in the United States

**DOI:** 10.3389/fpsyg.2025.1532636

**Published:** 2025-04-16

**Authors:** Catherine So-Kum Tang

**Affiliations:** Department of Counselling and Psychology, Hong Kong Shue Yan University, North Point, Hong Kong SAR, China

**Keywords:** work addiction, emotional dysregulation, addictive eating, physical health, workaholism

## Abstract

**Background and aims:**

This study explored the psychological mechanisms linking work addiction to poor physical health among full-time employees. The proposed serial multiple mediation model suggests that work addiction depletes employees’ ability to regulate emotions, leading to addictive eating as a coping mechanism, which creates a vicious cycle that severely impairs physical health.

**Methods:**

The sample consisted of 1,233 full-time employees (aged 25–65 years) in the United States, who completed an online survey assessing work addiction, emotion regulation deficits, addictive eating, and physical functioning.

**Results:**

Among all participants, the rates were 13.1% for food addiction, 9.7% for work addiction, and 3.5% for co-occurrence of food and work addiction. Compared to men, women reported a higher rate of food addiction, but the rates of work addiction were similar for both genders. The proposed model was tested using bootstrapping analysis, and the findings supported the serial multiple mediation model. The indirect effect of work addiction on poor physical health, mediated first by emotional dysregulation and then by addictive eating, was significant. Although the direct effect of work addiction on physical health was reduced when accounting for the mediators, it remained significant. The results also indicated that gender did not significantly moderate these relationships, showing consistent findings across men and women.

**Discussion:**

Work and eating addictions are common among full-time employees. Findings underscore the complex pathway through which work addiction exacerbates physical health problems via emotional and behavioral mechanisms.

**Conclusion:**

Work addiction impacts physical health both directly and indirectly, and is associated with emotional dysregulation and addictive eating. Implementing wellness programs that address emotional reactivity and provide nutrition education is essential to mitigate the negative health impacts of work addiction.

## 1 Introduction

Work dominates the adult lives of many individuals, driven by global competition and rapid technological advancements that make work accessible anytime, anywhere. Work addiction, or workaholism, refers to a compulsive and uncontrollable need to work incessantly (Andersen et al., 2023). It is often categorized as a behavioral addiction, sharing similarities with addiction to substances like alcohol or drug (Griffiths, 2005; Griffiths et al., 2018). Symptoms of work addiction include being excessively preoccupied with work (salience), using work to reduce emotional stress (mood modification), progressively working longer hours to the same emotional relief (tolerance), experiencing emotional and physical distress when unable to work (withdrawal), prioritizing work over personal, family, and social/or obligations (conflict); attempting unsuccessfully to control work hours (relapse), and facing harm or negative consequences due to excessive work (problems). A meta-analysis study reports approximately 14% of working individuals across countries may be addicted to work (Andersen et al., 2023). Research indicates work addiction is associated with various negative mental health outcomes, including depression, anxiety, obsessive-compulsive, and anankastic personality disorder (Dutheil et al., 2020). Workaholics often encounter sleep problems such as insomnia and daytime sleepiness, impacting their overall biopsychosocial health. This pattern of overworking leads to functional impairments, increased chronic illnesses, accident proneness, poor work performance, poor social relationships, and reduced quality of life (Kenyhercz et al., 2024; Vedoato et al., 2021).

This study aimed to uncover the psychological mechanisms linking work addiction to adverse health outcomes among full-time employees. The Conservation of Resources Theory (Hobfoll et al., 2018) and the Dual-Process Model of Coping (Stroebe and Schut, 1999) offer robust conceptual frameworks for understanding how work addiction can lead to compromised physical health. According to the COR Theory (Hobfoll et al., 2018), individuals strive to obtain, retain, and safeguard their resources, which encompass personal characteristics, circumstances, and energies. Work addiction involves an excessive investment of time and energy into work, often at the expense of other crucial aspects of life (Kenyhercz et al., 2024; Vedoato et al., 2021). This overinvestment in work can significantly deplete essential resources like time for rest, exercise, and social interactions, as well as emotional resources, making it harder for individuals to regulate their emotions effectively.

Emotional dysregulation refers to difficulties in managing and responding to emotional experiences (Agako et al., 2022). High levels of work addiction are found to be inversely related to self-regulation of emotions and psychological capital, as workaholics struggle to maintain positive emotions even during enjoyable activities (Menghini et al., 2023; Towch et al., 2024). Workaholic employees spend a substantial amount of time working, potentially depriving themselves of opportunities to recover from their excessive efforts. This emotional dysregulation stemming from overworking can drive individuals to further immerse themselves in work as a coping mechanism, creating a vicious cycle where emotional dysregulation exacerbates stress. The COR theory (Hobfoll et al., 2018) elucidates that the interplay between work addiction, emotional dysregulation, and impaired physical health is driven by the continual loss and depletion of valuable resources, perpetuating a downward spiral that worsens the overall health and well-being.

*Hypothesis 1*: Emotional dysregulation mediates the association between work addiction and physical health.

The Process Model of Coping (Stroebe and Schut, 1999, 2010) distinguishes between problem-focused and emotion-focused coping strategies. Problem-focused coping involves addressing stressors directly, while emotion-focused coping aims to manage the emotional response to stress. Workaholics, due to their emotional exhaustion and depleted resources, may struggle to engage in problem-focused coping and rely heavily on emotional-focused coping. However, this reliance can further deplete emotional resources, exacerbating emotional dysregulation. Addictive eating, or food addiction, entails a recurrent pattern of excessive food consumption despite negative consequences and loss of control over consumption (Hauck et al., 2020). This behavior involves using food as a coping strategy to manage stress, anxiety, or other unpleasant emotions (Ahmadkaraji et al., 2023; Fernández et al., 2024). Consuming high-calorie, highly palatable foods can offer temporary comfort and pleasure, potentially becoming habitual as individuals increasingly turn to food to manage their emotions rather than addressing the underlying issues (Ha and Lim, 2023; Hauck et al., 2020; Ljubičić et al., 2023).

*Hypothesis 2*: Emotional dysregulation mediates the relationship between work addiction and addictive eating.

Increasing evidence suggests that addictive eating is associated with health and mental health symptoms (Hoover et al., 2023; Horsager et al., 2021) and decreased psychosocial functioning (Lacroix and von Ranson, 2021). Within the context of work addiction, addictive eating can emerge as an ineffective coping strategy, providing temporary emotional relief. Over time, addictive eating can contribute to poor physical health, leading to issues like weight gain, obesity, and associated health problems such as diabetes and cardiovascular disease. The negative impact of declining physical health can exacerbate emotional distress, creating a vicious cycle where deteriorating health may increase stress and anxiety, prompting workaholic individuals to more addictive eating for quick and temporary relief.

*Hypothesis 3*: Addictive eating mediates the association between work addiction and physical health.

Emotion dysregulation plays a crucial role in addictive eating (Ahmadkaraji et al., 2023; Fernández et al., 2024; Ribeiro et al., 2023), driven by unsuccessful attempts to regulate emotions (Ahmadkaraji et al., 2023; Fernández et al., 2024). Emotional dysregulation and addictive eating, combined with the ongoing stress from overworking, significantly affect physical health, leading to poor sleep, heightened risk of chronic illnesses, and reduced overall well-being. Based on the Conservation of Resources Theory (Hobfoll et al., 2018) and the Dual-Process Model of Coping (Stroebe and Schut, 1999, 2010), a serial multiple mediation model is proposed to illustrate the sequence of psychological variables linking work addictions to poor physical health. The proposed model suggests that work addiction depletes emotional regulation abilities, with addictive eating serving as a maladaptive emotional regulation strategy to avoid, escape, or suppress negative emotions. Consequently, workaholic employees may experience poor physical health due to non-healthy eating habits. While many studies have reported negative associations between workaholism and physical health (e.g., Balducci et al., 2021; Engelbrecht et al., 2020; Menghini and Balducci, 2024; Wettstein et al., 2022), research exploring the sequence of mediators between work addition, emotional dysregulation, addictive eating, and physical health is relatively limited.

*Hypothesis 4*: Work addiction is associated with emotional dysregulation, which is linked to addictive eating, and both are related to impaired physical health. Specifically, the sequence of the association is work addiction → emotional dysregulation → addictive eating → poor physical health.

## 2 Method

### 2.1 Procedure

Full-time workers, who were at least 25 years old and resided in the United States, were recruited to this study. Full-time employment was defined as engaging in salaried work for an average of 35–40 h per week. A crowdsourcing Internet marketplace sent out nationwide email invites to recruit individuals that fit the inclusion criteria. Hyperlinks to the web survey were sent to those who consented to participate in the study. The web survey took about 15–20 min to complete. No personal identifiable information was asked. Upon completion of the survey, participants were given a code number to claim their participation fees from the Internet marketplace.

### 2.2 Participant characteristics

A total of 1,600 emails were sent nationwide to recruit individuals that fit the inclusion criteria. Among them, 1,233 individuals (671 women, 562 men) completed the web survey, yielding a response rate of 77%. Table 1 shows the descriptive data of participants’ demographic information. Participants’ ages ranged from 25 to 65, with the average age being 37.28 years old (SD = 9.16). Among all participants, about 60% were married and 71% completed at least tertiary/university education. Ethnicity of participants comprised of 82.6% Caucasian, 6.2% Asian, 6.1% African American, and 3.8% Hispanic or Latino. Participants worked in various types of industry, including hospitality (24.6%), professional/business (21.2%), education/health (18.7%), and information technology (18.5%).

**Table 1:** Demographic information (*N* = 1,233).

Demographic Information	*N* (%)
Age	
25–35 years	647 (52.5%)
36–45 years	349 (28.3%)
46–55 years	184 (14.9%)
56 years and older	53 (4.3%)
Gender	
Women	562 (45.6%)
Men	671 (54.4%)
Marital status	
Married/with partner	745 (60.4%)
Single/without partner	488 (39.6%)
Education	
Secondary school and lower	357 (29.0%)
Tertiary/University	876 (71.0%)
Type of industry	
Manufacturing/Construction/Transportation/Utilities	209 (17.0%)
Informational Technology	228 (18.5%)
Professional, Business, Finance	262 (21.2%)
Education and Health	230 (18.7%)
Leisure, Hospitality, Others	304 (24.6%)
Ethnicity	
Caucasian	1,019 (82.6%)
Asian	77 (6.2%)
African American	75 (6.1%)
Hispanic/Latino	47 (3.8%)
Others	15 (1.2%)

### 2.3 Measures

The Physical Functioning Scale (PFS) of the 36-Item Short Form Health Survey (SF-36) was utilized to evaluate participants’ general physical functioning over the past 2 weeks (Ware and Sherbourne, 1992). The PFS consists of 10 items that cover a hierarchical range of difficulties. It has demonstrated good internal consistency, with a Cronbach’s alpha greater than 0.80. The PFS has been employed as a standalone instrument in research to describe activity limitations among diverse groups. Each item on the PFS is scored based on the limitations perceived by the participants, using a scale from 1 (significant limitation) to 3 (no limitation at all). For this study, the internal consistency reliability was 0.93.

The severity of addictive eating was assessed by the 9-item Modified Yale Food Addiction Scale, based on the clinical criteria for substance dependence on the DSM-IV (mYFAS; Flint et al., 2014). It consists of nine items, with one item for assessing each of the seven symptoms and two items assessing the presence of a clinically significant impairment or distress during the past year. The seven symptoms include: food cravings, loss of control over excessive eating, increased food intake overtime, continued eating despite negative consequences, unsuccessful efforts to eat less, tolerance, and characteristic withdrawal symptoms. Participants indicated the frequency of their addictive eating behavior in the last 12 months with a 5-point scale ranging from 0 (never) to 4 (4 or more times per day or daily). Participants who scored at least 3 or more symptom items and 1 distress item on the mYFAS were classified as meeting the diagnostic threshold for food addiction (Flint et al., 2014). For the present study, the internal consistency reliability was 0.90.

The 18-item Difficulties in Emotion Regulation Scale Short Form (DERS-SF: Kaufman et al., 2016) was used to assess deficits in emotion regulation. The DERS-SF includes six subscales, namely: (1) non-acceptance of emotional responses or denial of distress, (2) difficulties in engaging goal-directed behavior while experiencing negative emotions, (3) difficulties in impulse control when upset, (4) lack of emotional awareness, (5) beliefs that there is little one can do to regulate one’s emotions effectively, and (6) lack of emotional clarity. Sample items of the scale include “You have difficulty making sense out of your feelings” and “It takes you a long time to feel better when you are upset.” Participates were asked to indicate how often the items apply to themselves in the past year, with responses ranging from 1 as “Almost never” to 5 “Almost always.” For this study, items were recoded such that emotion dysregulation was represented by higher scores on the items. The internal reliability of this scale for this sample was 0.89.

Work addiction was measured by the 7-item Bergin Work Addiction Scale (Andreassen et al., 2012). This scale consists of items to assess individuals’ perception of compulsive and excessive work behavior using addiction criteria (i.e., salience, tolerance, mood modification, relapse, withdrawal, conflict, and problems) experienced during the past year. Sample items include: “spent much more time working than initially intended,” “been told by others to cut down on work without listening to them,” and “becoming stressed if prohibited from working.” Each item is answered on a 5-point Likert scale ranging from 1 (never) to 5 (always). Higher scores indicate more severe workaholism symptoms. Participants who scored 4 (often) or 5 (always) on four out of seven items of the Bergin Work Addiction Scale were classified as manifesting work addiction (Andreassen et al., 2012). For the present study, the internal consistency reliability was 0.86.

Participants were also asked to indicate their age, gender, marital status, educational attainment, type of industry that they currently engaged in, and working hours per week.

### 2.4 Statistical analysis

**Figure 1:**
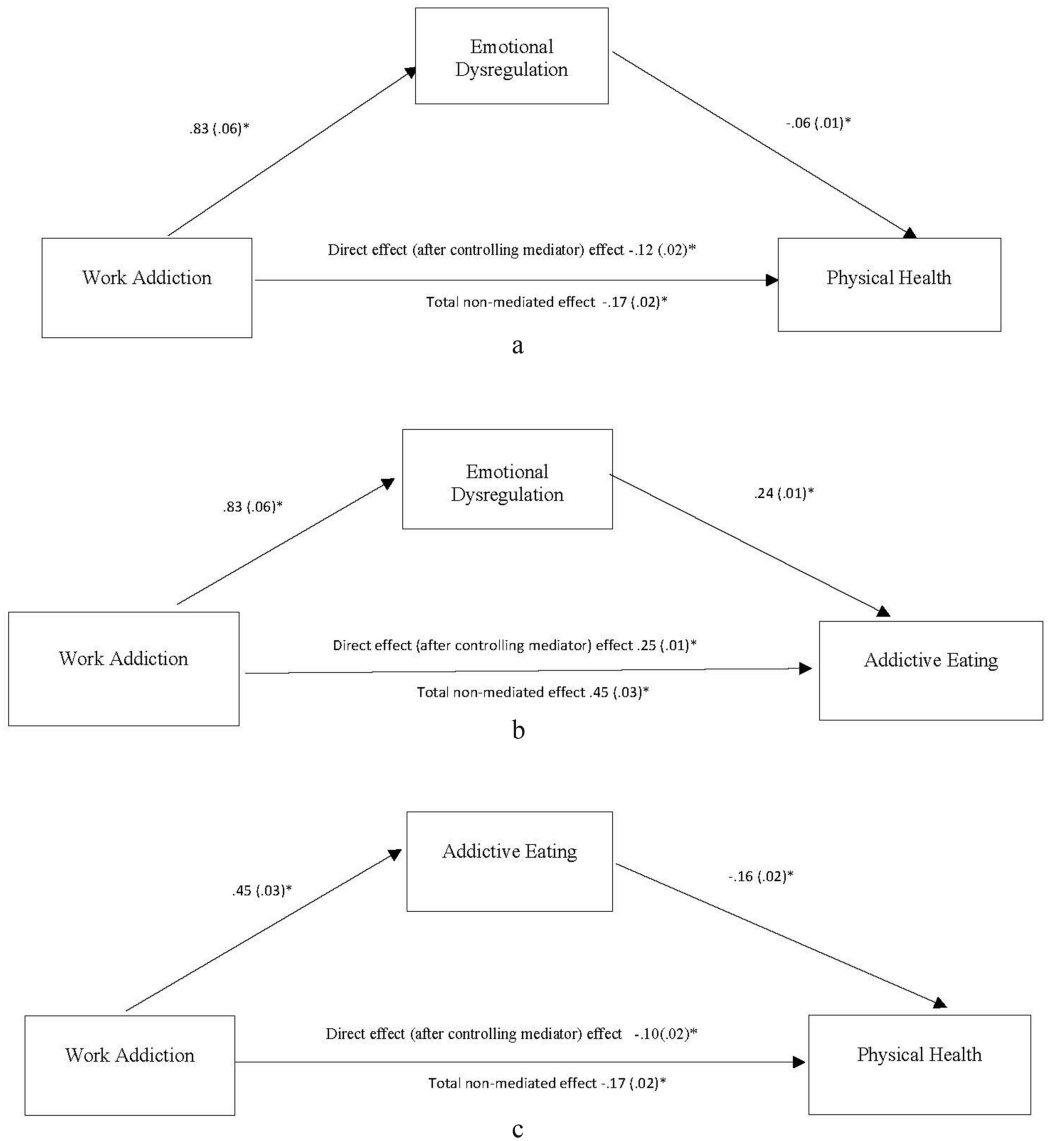
Summary of single mediation models. **(a)** Indirect effect of work addiction → emotional dysregulation → physical health estimated to be −0.05, 95% CI = −0.06 to −0.03, *p* < 0.05. **(b)** Indirect effect of work addiction → emotional dysregulation → addictive eating estimated to be 0.20, 95% CI = 0.06 to 0.24, *p* < 0.05. **(c)** Indirect effect of work addiction → addictive eating → physical health estimated to be −0.07, 95% CI = −0.09 to −0.05, *p* < 0.05. Values presented in the figures are coefficients and standard errors. *p < 0.01.

**Figure 2:**
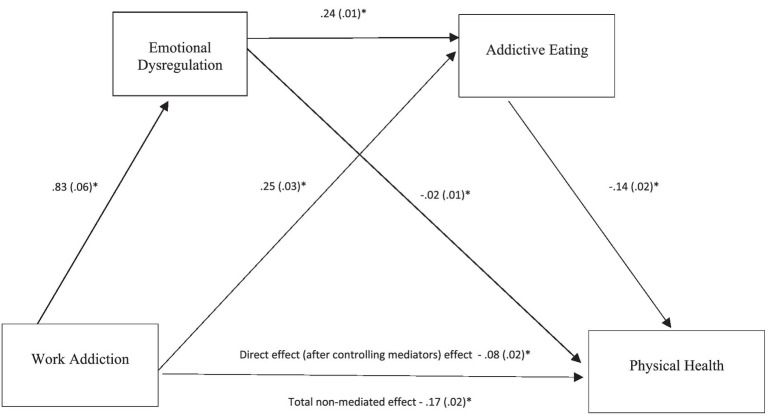
A serial multiple mediation model of work addiction-emotion dysregulation-addictive eating-health association. Total indirect effect of the serial multiple mediation estimated −0.09, 95% CI = 11 to −0.06, *p* < 0.05. Indirect effect of work addiction → emotional dysregulation → physical health estimated to be −0.02, 95% CI = −0.04 to 0.01, *p* > 0.05. Indirect effect of work addiction → addictive eating → physical health estimated to be −0.04, 95% CI = 0.05 to −0.02 to −0.02, *p* < 0.05. Indirect effect of work addiction → emotional dysregulation → addictive eating → physical health estimated to be −0.03, 95% CI from −0.04 to −0.02, *p* < 0.05. Values presented in the figures are coefficients and standard errors *p < 0.01.

The IBM SPSS 27.0 computer software was used for statistical analyses. Descriptive analyses of sample characteristics and major variables were conducted (Table 1). Bivariate correlations were computed to examine associations between two variables (Table 2). A multiple regression analysis was performed to identify the best predictors for physical health. The SPSS PROCESS macros version 3 (Hayes, 2017) was used for bootstrapping analyses to determine the significance of mediators. The indirect, direct, and total effects of work addiction on physical health via the two mediators (emotional dysregulation and addictive eating) were determined. An effect was considered as significant if its 95% bootstrap confidence interval from 5,000 bootstrap samples does not include zero. Three single mediation models were first conducted to test the influences of work addiction, addictive eating, and emotion dysregulation on physical health (Figure 1). The proposed serial multiple mediation model that specified work addiction → emotional dysregulation → addictive eating → physical health was then tested (Table 3 and Figure 2).

**Table 2:** Bivariate correlations of demographic and major variables.

Variables	1	2	3	4	5	6	7
1. Gender (1 = Female, 2 = male)	1						
2. Age	−0.09*	1					
3. Working hours per week	0.02	0.14**	1				
4. Work addiction	−0.03	−0.02	0.25**	1			
5. Emotion dysregulation	0.03	−0.12**	0.01	0.38**	1		
6. Addictive eating	−0.10**	0.01	0.03	0.34**	0.52**	1	
7. Physical health	0.08*	−0.11**	−0.04	−0.24**	−0.24**	−0.33**	1
Internal Consistency	–	–	–	0.86	0.89	0.90	0.93
Mean	–	37.28	45.36	16.29	36.78	7.89	28.01
Standard Deviation	–	9.16	8.24	5.55	12.14	6.70	3.84

*p < 0.01, **p < 0.001.

**Table 3:** Comparisons of the indirect effects of mediation models.

Mediation Models	Standardized indirect effect	Indirect effect	BootSE	Bootstrapping 95% CI	
				Lower	Upper
Indirect effects of single mediation models					
H1: Work addiction → emotional dysregulation → physical health	−0.01*	−0.05	0.01	−0.06	−0.03
H2: Work addiction → emotional dysregulation → addictive eating	0.17*	0.20	0.02	0.16	0.24
H3: Work addiction → addictive eating → physical health	−0.10*	−0.07	0.01	−0.09	−0.05
**Total indirect effects of serial mediation model** ^a^	−0.12*	**−0.09**	**0.01**	**−0.11**	**−0.06**
Indirect effect of Work addiction → emotional dysregulation → physical health	−0.03	−0.02	0.01	−0.04	0.01
Indirect effect of Work addiction → addictive eating → physical health	−0.05*	−0.04	0.01	−0.05	−0.02
H4: Indirect effect of Work addiction → emotional dysregulation → addictive eating → physical health	−0.04*	−0.03	0.01	−0.04	−0.02

^a^Total indirect effects of the proposed serial mediation model. *Significant indirect effects *p* < 0.05.

### 2.5 Ethics statement

This study was approved by the Institutional Ethics Review Board of the affiliated university of the author at the time when data was collected. All participants were informed about the study and all provided informed consent.

## 3 Results

### 3.1 Preliminary analyses

Independent *t*-tests revealed that women compared to men were older (Means = 38.21 and 36.51, respectively; *t* = 3.26, *p* = 0.001), exhibited more frequent addictive eating behaviors (Means = 8.60 and 7.29, respectively; *t* = 3.43, *p* = 0.001), and reported poorer physical health (Means = 27.67 and 28.30, respectively; *t* = −2.85, *p* = 0.005). Bivariate correlations indicated that physical health was negatively associated with age (*r* = −0.11, *p* < 0.001), work addiction (*r* = −0.24, *p* < 0.001), emotional dysregulation (*r* = −0.24, *p* < 0.001), and addictive eating (*r* = −0.33, *p* < 0.001). There were no significant differences in physical health among participants with varying marital status, ethnicity, types of industry, and working hours (*p*s > 0.05). A multiple regression analysis, incorporating demographic information (age, gender, marital status, ethnicity, education, types of industry, and working hours) and psychological variables (workaholism, emotional dysregulation, and addictive eating), was conducted to predict physical health. The results indicated that the best predictors of physical health were addictive eating (*β* = −0.23, *p* = 0.000), work addiction (*β* = −0.13, *p* = 0.000), emotional dysregulation (*β* = −0.08, *p* = 0.009), and female gender (*β* = 0.07, *p* = 0.009).

The prevalence rates for work and food addiction among all participants were 9.7 and 13.1%, respectively. Women exhibited a higher rate of food addiction compared to men (16% vs. 10.6%; *X*^2^ = 7.95, *p* = 0.001). The co-occurrence of food and work addiction was observed in 3.5% of participants. However, no significant differences were found between men and women in the rates of work addiction or the co-occurrence of food and work addiction (*p* > 0.05).

### 3.2 Testing single mediation models

Three single mediation analyses were performed to test the first three hypotheses (Model 4 of the SPSS PROCESS procedure, Figure 1). All indirect effects were summarized in Table 3. For the work addiction-emotion regulation-physical health mediation analysis, the direct effect of work addiction on physical health after controlling for emotional dysregulation was reduced but remained significant (*β* = −0.12, *t* = −5.97, *p* = 0.000, 95% CI of −0.17 to −0.08). The indirect effect of work addiction on physical health through emotional dysregulation was significant and estimated to be −0.05 with a 95% CI of −0.06 to −0.3. Thus, Hypothesis 1 was partially supported, and emotional dysregulation was a significant mediator between workaholism and poor physical health.

For work addiction-emotional dysregulation-addictive eating mediation analysis, the direct effect of work addiction on addictive eating after controlling for emotional dysregulation was reduced but remained significant (*β* = 0.25, *t* = 8.08, *p* = 0.000, with a 95% CI 0.19 to 0.31). The indirect effect of work addiction on addictive eating through emotional dysregulation was significant and estimated to be 0.20 with a 95% CI of 0.16 to 0.24. Hypothesis 2 was partially supported.

For the work addiction-addictive eating-health mediation analysis, the direct effect of work addiction on physical health after controlling for addictive eating was reduced but remained significant (*β* = −0.10, *t* = −4.83, *p* = 0.000, with a 95% CI of −0.14 to −0.06). The indirect effect of work addiction on physical health through addictive eating was significant and estimated to be −0.07 with a 95% CI of −0.09 to 0.5. Thus, Hypothesis 3 was partially supported, and addictive eating was a significant mediator between work addiction and physical health.

### 3.3 Testing the proposed serial multiple mediation models

Bootstrapping results partially supported the proposed serial multiple mediation model, i.e., work addiction → emotional dysregulation → addictive eating → physical health (Model 6 of the SPSS PROCESS procedure, Figure 2). The direct effect of work addiction on physical health through the two mediators was reduced but remained significant (*β* = −0.08, *t* = −4.22, *p* = 0.000, 95% CI = −0.13 to −0.05). The indirect effect of work addiction first through emotional dysregulation then through addictive eating was significant and estimated to be −0.03 with a 95% bootstrap CI of −0.04 to −0.02. Thus, Hypothesis 4 was supported. It was further noted that within the serial mediation framework, the indirect effect of work addiction on physical health through emotional dysregulation became non-significant. However, the indirect effect of work addiction on physical health through addictive eating remained significant and estimated to be −0.04 with a 95% bootstrap CI of −0.05 to −0.02.

Gender was identified by the multiple regression analysis as one of the predictors, hence, a follow-up analysis of the proposed serial multiple mediation model was performed with gender as the moderator. Results showed that gender was not a significant moderator (*β* = 0.09, *p* = 0.07, 95% CI = −0.01 to 0.17). In other words, the proposed model and its mediation pathways were consistent across men and women.

## 4 Discussion

The present study found that approximately 13.1% of surveyed full-time employees reported food addiction, 9.7% reported work addiction, and about 3.5% reported both food and work addiction. These prevalence rates align with existing literature (Andersen et al., 2023; Oliveira et al., 2021), indicating the widespread nature of these behaviors among working adults. The co-occurrence of work and eating addictions suggests that these behaviors may share common underlying mechanisms and stressors, such as chronic stress, emotional dysregulation, and maladaptive coping strategies. This co-occurrence supports the Conservation of Resources Theory (Hobfoll et al., 2018), which posits that individuals strive to retain and protect their resources, and when these resources are threatened or depleted, stress and maladaptive behaviors, like food addiction, will emerge. Additionally, the findings are also consistent with the Dual-Process Model of Coping (Stroebe and Schut, 1999, 2010), which suggests that individuals use both problem-focused and emotion-focused coping strategies to manage stress. In the context of work addiction, the inability to manage emotions effectively can lead to heavy reliance on addictive eating as an emotion-focused coping strategy, further impairing physical health. Other research also found that work addiction is significantly related to physical health (Balducci et al., 2021; Engelbrecht et al., 2020; Menghini and Balducci, 2024; Wettstein et al., 2022), emotional dysregulation (Menghini et al., 2023; Towch et al., 2024) and addictive eating (Fekih-Romdhane et al., 2024).

More importantly, the results support the proposed serial multiple mediation model, demonstrating the significant indirect effects of work addiction on poor physical health, mediated first by emotion dysregulation and subsequently by addictive behaviors. This finding underscores the complex pathway through which work addiction exacerbates physical health problems via emotional and behavioral mechanisms. Addictive eating emerges as the most proximal psychological factor linking work addiction to physical health, consistent with previous literature suggesting addictive eating is related to higher body mass index (Hoover et al., 2023), impaired physical functioning, and lower health-related quality of life (Brytek-Matera et al., 2021; Menghini and Balducci, 2024; Minhas et al., 2021). Addictive eating behaviors are often driven by the need to mitigate negative emotions and regulate negative affect state. When individuals struggle with emotion regulation, they may resort to addictive eating as a coping strategy (Ahmadkaraji et al., 2023; Fernández et al., 2024), further impacting their physical health (Brytek-Matera et al., 2021; Balducci et al., 2021; Engelbrecht et al., 2020; Menghini and Balducci, 2024; Minhas et al., 2021; Wettstein et al., 2022). Workaholic employees, who often experience difficulties in regulating emotions (Menghini et al., 2023; Towch et al., 2024), may turn to addictive eating to manage their emotional states (Fekih-Romdhane et al., 2024), thereby exacerbating physical health issues.

Results showed that the rates of work addiction and co-occurrence with addictive eating among men and women did not differ significantly. This reflects shared influences and stressors faced by both genders in their professional life. Work environments reward productivity and dedication irrespective of gender, reinforcing the compulsion to work excessively. Furthermore, technological advancements have made work more accessible, blurring the boundaries between personal and professional life for men and women. Consequently, the rates of co-occurring work and eating addiction are similar across genders due to comparable levels of work-related stress and societal pressures. However, women reported higher rates of addictive eating and poorer physical health compared to men. This disparity may stem from gender-specific emotional reactivity, stressors, and coping mechanisms (Cavagnis et al., 2023; Mengelkoch and Slavich, 2024). Biologically, hormonal differences, particularly fluctuations in estrogen and progesterone, affect mood and emotional responses (Mengelkoch and Slavich, 2024). Women often face additional societal and cultural pressures, such as balancing work and family responsibilities, leading to higher level of stress and emotional exhaustion (Cavagnis et al., 2023). They are also socialized to be more in touch with their emotions and express them more freely, leading to greater emotional awareness and reactivity (Givon et al., 2023). Furthermore, women are more likely to experience psychiatric comorbidities and greater exposure to sexual traumas (Lake et al., 2024; Smari et al., 2023), which can further exacerbate stress. This heightened stress may drive them toward addictive eating as a coping strategy, exacerbating physical health issues. Eating is reinforced by societal norms that often associate food with comfort and emotional solace, making addictive eating a prevalent coping mechanism among women. Despite these differences in coping strategies and health outcomes, gender did not significantly moderate the relationships among variables as in the proposed serial multiple mediation model. This suggests that the underlying mechanisms linking work addiction and poor health are similar across genders.

This study has several limitations that need to be acknowledged. The cross-sectional design limits the ability to infer causality between work addiction, emotional dysregulation, addictive eating, and poor physical health. The reliance on self-reported data introduces potential biases such as social desirability and recall bias, which may affect the accuracy of the findings. The sampling method, which involved full-time employees completing an online survey, may exclude individuals without internet access or those less comfortable with technology, thereby limiting the generalizability of the results. The study’s focus on full-time employees in the United States may not represent the experiences of individuals from different countries, cultural backgrounds, or employment types. This study focuses on individual attributes without considering family, social, and work environment factors that may also influence the relationship between work addiction and physical health.

### 4.1 Research and practical implications

Future research on the relationship should include conducting longitudinal studies that can provide insight into the long-term effects and establish causal relationships among variables. Including diverse samples in terms of age, socioeconomic status, and cultural backgrounds ensures that findings are generalizable across different populations. Developing and testing targeted interventions that address both work addiction and addictive eating, such as stress management programs and emotional regulation training, can help identify effective strategies. Additionally, conducting gender-specific analyses can uncover unique differences in coping mechanisms and health outcomes, allowing for more tailored interventions. Integrating biopsychosocial models will enable researchers to explore the complex interplay of biological, psychological, and social factors, leading to more holistic and effective solutions. By adopting these approaches, future research can provide deeper insights and improve interventions to enhance overall well-being.

The practical implications of the findings underscore the need for comprehensive and inclusive workplace interventions to address work addiction and its associated behaviors, like addictive eating. By recognizing the interconnected nature of these issues, organizations should foster a supportive work environment that promotes work-life balance, provides resources for stress management, and encourages healthy coping strategies. Additionally, incorporating wellness programs that address emotional reactivity and offer nutrition education can help mitigate the negative health impacts of work addiction, emotional dysregulation, and addictive eating. Recognizing that women report higher levels of addictive eating and poorer physical health, additional support tailored to their unique stressors and coping strategies may be necessary. This approach can help ensure that all employees receive the support they need to maintain both their mental and physical health. Overall, these findings highlight the importance of holistic approaches to employee well-being that consider both psychological and physical health factors.

## 5 Conclusion

This study offers preliminary findings on the psychological mechanisms affecting work addicts’ physical health. Work addiction significantly impacts physical health both directly and indirectly, and is associated with emotional dysregulation and addictive eating. Implementing wellness programs that address emotional reactivity and provide nutrition education is essential to mitigate the negative health impacts of work addiction.

## Data Availability

The raw data supporting the conclusions of this article will be made available by the authors, without undue reservation.
